# An Incidental Finding of Pleomorphic Adenoma of the Minor Salivary Glands in the Skin Area of the Lower Lip

**Published:** 2014-10-22

**Authors:** Aditya Sood, Stella Chung, Ramazi O. Datiashvili

**Affiliations:** Division of Plastic Surgery, Department of Surgery, Rutgers University/New Jersey Medical School, Newark, NJ

**Keywords:** pleomorphic adenoma, salivary gland tumors, minor salivary gland, facial mass, facial pain

## Abstract

Salivary gland tumors are uncommonly seen and account for less than 3% of the head and neck tumors. Pleomorphic adenoma is a well-described benign tumor of the salivary glands, originating from myoepithelial and intercalated duct cells. It is most commonly found in major salivary glands. We present a rare and unusual case of pleomorphic adenoma of the minor salivary glands in the lower lip. The tumor was diagnosed upon excision of 1.5 × 1.2 cm^2^ well-circumscribed nodule at the junction of the lower lip and chin in a 46-year-old man. The histopathological analysis confirmed presence of an epithelial salivary gland tumor with islands of plasmacytoid cells, and duct-like structures within a variable and mixed stroma.

Salivary gland tumors are rare and seen in less than 3% of all head and neck tumors.^1^ They are most commonly found in the parotid gland, followed by submandibular gland and minor salivary glands.^2^ Tumors of minor salivary glands comprise 15% to 20% of all salivary gland neoplasms. The majority of them are benign; however, they have a higher likelihood of being malignant. Minor salivary gland tumors are rare neoplasms of the upper aerodigestive tract.^3^ Pleomorphic adenoma (PA) comprises 40% to 72% of all minor salivary gland tumors; however, benign PA of minor salivary glands arising de novo is very rare.^1^^,^^4^^-^10 The annual incidence of PA is estimated to be approximately 2 to 3.5 cases per 100,000 populations.^11^ PA is a well-described benign tumor of the salivary glands, originating from myoepithelial and intercalated duct cells. It is commonly found in major salivary glands: approximately 80% of the time in the parotid gland, 10% in the submandibular gland, and 10% in the minor salivary glands. Patients are often asymptomatic, and lesions may be found incidentally. Complete excision is the criterion standard for treatment and essential for preventing recurrence and malignant transformation. In this case report, we present an incidental finding of PA of the minor salivary glands in the lower lip.

## METHODS

Our case describes an unusual soft tissue lesion noted 1 cm below the left lower border of the lower lip in a 46-year-old Asian man. The patient complained of occasional pain from the site. The lesion was growing slowly over the past 2 years. Externally, the lesion appeared to be approximately 1.5 × 1.2 cm^2^ in dimensions, raised, rubbery, firm, mobile, and discreet. No punctum or skin extension was noted. The lesion was considered as benign from clinical examination, and the differential diagnosis was considered between lipoma versus sebaceous cyst.

## RESULTS

**Figure 1:**
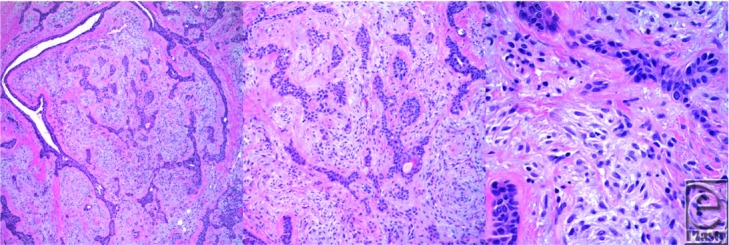
**(*a*)-(*c*).** Histopathological images in 10×, 20×, and 60×, respectively, revealing an epithelial salivary gland tumor, islands of plasmacytoid cells, and duct-like structures within a variable and mixed stroma.

Excision of the lesion was performed via transverse skin incision. Surgical extrication revealed a well-circumscribed, rubbery nodule; it was located within the subcutaneous tissue plane. It measured approximately 1.3 × 1.1 × 0.9 cm^3^. Final histopathology returned 1 week postoperatively revealing an epithelial salivary gland tumor with islands of plasmacytoid cells, and duct-like structures within a variable and mixed stroma indicative of a PA (Figs 1a-1c). Complete excision of the lesion was confirmed with histopathological margins. Post-operatively, there were no complications, the incision healed by primary intent. The patient was given the pertinent counseling and instructions including follow-up.

## DISCUSSION

Salivary gland tumors make up less than 1% of all tumors and 3% to 5% of all head and neck neoplasms. Minor salivary gland tumor is a rare neoplasm of upper aerodigestive tract and comprises 15% to 20% of all salivary gland tumors.^3^^-^9 They are most commonly encountered in the palate but also arise in the oral cavity, paranasal sinuses, nasopharynx, and larynx. Salivary gland neoplasm can occur at any age; the highest incidence is in the fourth decade of life for benign lesions and between fifth and seventh decades of life for malignant tumors.^10^ Classically, these lesions have been reported to be more frequent in women, though the proportion varies according to the histological type of tumor.^10^ The most common type of minor salivary gland tumors is PA, comprising 40% to 72% of all minor salivary gland tumors.^5^^-^10 According to 2005 World Health Organization classification of salivary gland tumors, PA is an epithelial type tumor that is benign. On the basis of a population-based study in the United States, PA is extremely rare with an estimated annual age-adjusted incidence varying from 1 in 30,000 to 50,000 persons.^11^

The name “Pleomorphic Adenoma” was suggested by Willis due to its unique histopathology characteristics.^12^ Pleomorphic adenoma is a morphologically complex entity as the epithelial and myoepithelial tumor cells can differentiate into fibrous, hyalinized, mucoid, myxoid, chondroid, osseous, or lipomatous tissue.^13^ Classically, PA is an encapsulated tumor; with nonmalignant lateral extensions into the capsule commonly encountered.^14^ The lesion most often arises in the superficial lobe of the parotid gland but may also be seen in the submandibular and minor salivary glands.^15^ Our patient's histopathological analysis reported an epithelial salivary gland tumor, islands of plasmacytoid cells, and duct-like structures within a variable and mixed stroma.

Signs and symptoms vary according to the location and size of tumor.^16^ Typically, PA presents as a slowly growing, mobile, and discrete nodule. Patients are often asymptomatic and the lesion is an incidental finding during unrelated medical or dental visit, as seen in our patient. As the tumor grows, later findings include ulceration, pain, paresthesia, dysphagia, speech impairment, referred otalgia, or even, rarely, facial paralysis secondary to extrinsic compression of the seventh cranial nerve.^17^^-^19 Pleomorphic adenoma is more often seen in women in the fourth to sixth decade of their life, and the etiology is unknown.^20^^,^^21^

Physical examination alone does not suffice in distinguishing benign tumors of minor salivary gland from malignant types. Therefore a biopsy is indicated, which should be performed to ensure the lesion is benign. Preoperative imaging, incisional or excisional biopsy, or fine-needle aspiration biopsy must be done prior to surgery.^17^ In our case, due to relatively small size of the tumor, excisional biopsy was performed to confirm the diagnosis of a benign epithelioid tumor, that is, PA.

The criterion standard for treatment of PA is complete excision with an adequate margin. Incomplete resection of all minor salivary gland tumors leads to recurrence and yields difficulty in future operations.^22^ Pleomorphic adenoma relapse is estimated to occur in 5% to 30% of cases and is almost always a result of incomplete surgical resection that especially manifests in the form of multiple foci and may be found to be aggressive.^10^ Pleomorphic adenoma also carries a high rate of implantability; therefore, caution must be exercised not to rupture the capsule or leave residual tumor cells behind including the extensions into the surrounding tissues.^23^ Carcinoma ex-PA arises from untreated PA and occurs up to 3% of minor salivary gland tumors.^18^ Treatment of malignant tumors may require palatectomy for palatal lesions, removal of the buccinators muscle for cheek lesions, and removal of the maxilla or mandible if they are involved.^22^ Extended resection may require reconstructive surgery by local flap or obturator prosthesis.^17^

The prognosis of PA after complete excision is excellent. According to a meta-analysis of parotid tumors, PA recurrence rate was estimated to be 3.4% after 5 years and 6.8% after 10 years.^24^

## CONCLUSIONS

Pleomorphic adenomas are frequently asymptomatic benign lesions, patients may not be aware of their existence, or the tumor is discovered incidentally in many cases. Rarely may they present in the minor salivary glands with unusual location, that is, the lower lip, as in the presented case. The treatment of choice after an appropriate workup is a complete excision. Follow-up at least after 5 years is warranted to rule out recurrence.
